# Lipocalin-2 (Lcn-2) Attenuates Polymicrobial Sepsis with LPS Preconditioning (LPS Tolerance) in FcGRIIb Deficient Lupus Mice

**DOI:** 10.3390/cells8091064

**Published:** 2019-09-11

**Authors:** Thunnicha Ondee, Joseph Gillen, Peerapat Visitchanakun, Poorichaya Somparn, Jiraphorn Issara-Amphorn, Cong Dang Phi, Wiwat Chancharoenthana, Devikala Gurusamy, Aleksandra Nita-Lazar, Asada Leelahavanichkul

**Affiliations:** 1 Department of Preventive and Social Medicine, Faculty of Medicine, Chulalongkorn University, Bangkok 10330, Thailand; 2 Functional Cellular Networks Section, Laboratory of Immune System Biology, National Institute of Allergy and Infectious Diseases, National Institutes of Health, Bethesda, MD 20892-1892, USA; 3 Department of Microbiology, Faculty of Medicine, Chulalongkorn University, Bangkok 10330, Thailand; 4 Center of Excellence in Systems Biology, Research affairs, Faculty of Medicine, Chulalongkorn University, Bangkok 10330, Thailand; 5 Surgery Branch, National Cancer Institute, National Institutes of Health (NIH), Bethesda, MD 20892, USA; 6 Translational Research in Inflammation and Immunology Research Unit (TRIRU), Department of Microbiology, Chulalongkorn University, Bangkok 10330, Thailand

**Keywords:** Fc gamma receptor IIb deficient macrophages, lupus, Lipocalin 2, neutrophil gelatinase-associated lipocalin (NGAL)

## Abstract

In patients with active lupus, spontaneous endotoxemia and possibly tolerance to lipopolysaccharide (LPS) is a potentially adverse complication. Similarly, previous reports have demonstrated that FcGRIIb deficient mice (FcGRIIb-/-; a lupus mouse model) are susceptible to LPS tolerance-induced decreased cytokine responses that inadequate for the organismal control. Thus, understanding the relationship between FcGRIIb and LPS tolerance could improve the therapeutic strategy for lupus. LPS tolerance can be induced through sequential LPS stimulations in either cells or a model organism. In RAW264.7 (a mouse macrophage cell-line), sequential LPS stimulation induced the secretion of Lipocalin-2 (Lcn-2) despite reduced cytokine secretion and severe energy depletion, as measured by the extracellular flux analysis, typical of LPS tolerance. In contrast, treatment with recombinant Lcn-2 (rLcn-2) attenuated LPS tolerance, as shown by an increase in secreted cytokines and altered macrophage polarization toward M1 (increased *iNOS* and *TNF-α*) in RAW264.7 cells. These results suggest a role of Lcn-2 in LPS tolerance attenuation. In bone marrow derived macrophages, Lcn-2 level was similar in LPS tolerant FcGRIIb-/- and wild-type (WT) cells despite the increased LPS tolerance of FcGRIIb-/- cells, suggesting relatively low basal levels of Lcn-2 produced in FcGRIIb-/- cells. In addition, attenuation of LPS tolerance effectuated by granulocyte-monocyte colony stimulating factor (GM-CSF) reduced Lcn-2 in both cell types, implying an inverse correlation between Lcn-2 and the severity of LPS tolerance. Consequently, rLcn-2 improved LPS tolerance only in FcGRIIb-/- macrophages and attenuated disease severity of cecal ligation and puncture (CLP) sepsis pre-conditioning with sequential LPS injection (LPS-CLP model) only in FcGRIIb-/- mice, but not in WT mice. To summarize, inadequate Lcn-2 production in FcGRIIb-/- macrophage might, at least in part, be responsible for the inordinate LPS tolerance compared with WT cells. Additionally, supplementation of rLcn-2 attenuates LPS tolerance in FcGRIIb-/- macrophages in vitro, and in FcGRIIb-/- mice with LPS-CLP sepsis in vivo. In conclusion, Lcn-2 secreted by macrophages is possibly an autocrine signal to counter the reduced cytokine secretion in LPS tolerance.

## 1. Introduction

The functional defect of Fc gamma receptor IIb (FcGRIIb), the only inhibitory receptor among FcGR family, is one of the genetic causes of systemic lupus erythematosus (SLE) [1,2,3]. FcGRIIb-/- mice have been used as a representative lupus model for Asian populations due to the high prevalence of the dysfunction-polymorphism of the FcGRIIb gene [4]. Active lupus induces spontaneous endotoxemia [5,6], which in turn, may cause lipopolysaccharide (LPS) exhaustion [7,8,9,10] resulting in increased infection susceptibility [11]. Although the effective organismal control due to the loss of inhibitory signal has been demonstrated in FcGRIIb-/- mice [3,12,13], the extreme exhaustion after repeated stimulations or infections has also been mentioned as a possible cause [11,14].

The increased infection susceptibility during the immune exhaustion state is well described [15,16,17] and possibly correlates with the increased infections in lupus [18,19]. As such, lethal response against severe infection, known as “sepsis”, is prominent in lupus [20]. Endotoxemia is also common in sepsis due to the translocation of LPS from gut into blood circulation due to gut permeability defect [21]. Hence, chronic LPS stimulation in a clinical situation is possible and the adaptation of innate immune response (mostly mononuclear cells) toward several LPS stimulations, referred to as “LPS tolerance”, is well established. Interestingly, LPS tolerance is characterized by a diminished respond against LPS after an initial LPS exposure, unfortunately, without specific molecular cell surface-markers [7].

While LPS tolerance protects the host from a lethal dose of LPS (by decreasing cytokine responses), decreased cytokine secretion in LPS tolerance impairs organismal level control mechanisms [11]. The underlying mechanisms of LPS tolerance are still not completely known but possibly rely on Toll-like-receptor 4 (TLR4), microRNA and cell energy depletion [7] and may be different between FcGRIIb-/- and wild-type (WT) mice. Indeed, we have recently demonstrated the higher activity of protein kinase C-β Type II in FcGRIIb-/- macrophages with LPS tolerance compared with WT [14]. Because the secreted proteins might be responsible either for the compensation or activation of LPS tolerance, secretome analysis was performed and the interesting protein, lipocalin2 (Lcn-2 or NGAL) was studied further both in vitro and in vivo.

## 2. Materials and Methods

### 2.1. Animals and Animal Model

The animal study protocols were approved by Faculty of Medicine, Chulalongkorn University followed the National Institutes of Health (NIH) criteria (SST 002/2559, May 2016). The FcGRIIb-/- mice (C57BL/6 background) were provided by Dr. Silvia M. Bolland (National Institute of Allergy and Infectious Diseases, NIH, Bethesda, MD, USA). All the other mice were purchased from the National Laboratory Animal Center, Nakornpathom, Thailand. Eight week old female C57BL/6 mice were used in all experiments. Cecal ligation and puncture (CLP) surgery preconditioning with LPS tolerance (LPS-CLP) model was performed following a previously described protocol [11]. Briefly, endotoxin (LPS) from *Escherichia coli* 026:B6 (Sigma-Aldrich, St. Louis, USA) was administered intraperitoneally in two separate doses at 0.8 mg/kg and 5 days later at 4 mg/kg, followed by CLP surgery 12 h later with the ligation at 10 mm from the cecal tip and twice-puncture with a 25-gauge needle. Fentanyl at 0.03 mg/kg in 200 µL NSS was administered immediately post-operation and 6 h later. Mice were sacrificed at 24 h post-CLP for serum collection through cardiac puncture under isoflurane anesthesia. In the survival analysis, mice were observed for 96 h post-CLP before sacrifice. Serum creatinine and alanine transaminase were measured by QuantiChrom Creatinine Assay (DICT-500; Bioassay, Hayward, CA, USA) and EnzyChrom ALT assay (EALT-100, BioAssay), respectively. Serum cytokines were measured by Quantikine (R&D systems, Minneapolis, MN, USA). To test the effectiveness of recombinant Lipocalin-2 (rLcn-2), intravenous administration of rLcn-2 (R&D systems) at 6 mg/kg through tail vein before CLP operation was performed.

### 2.2. Secretome-Proteomic Analysis of LPS Tolerance in RAW264.7 Cells

**Figure 1:**
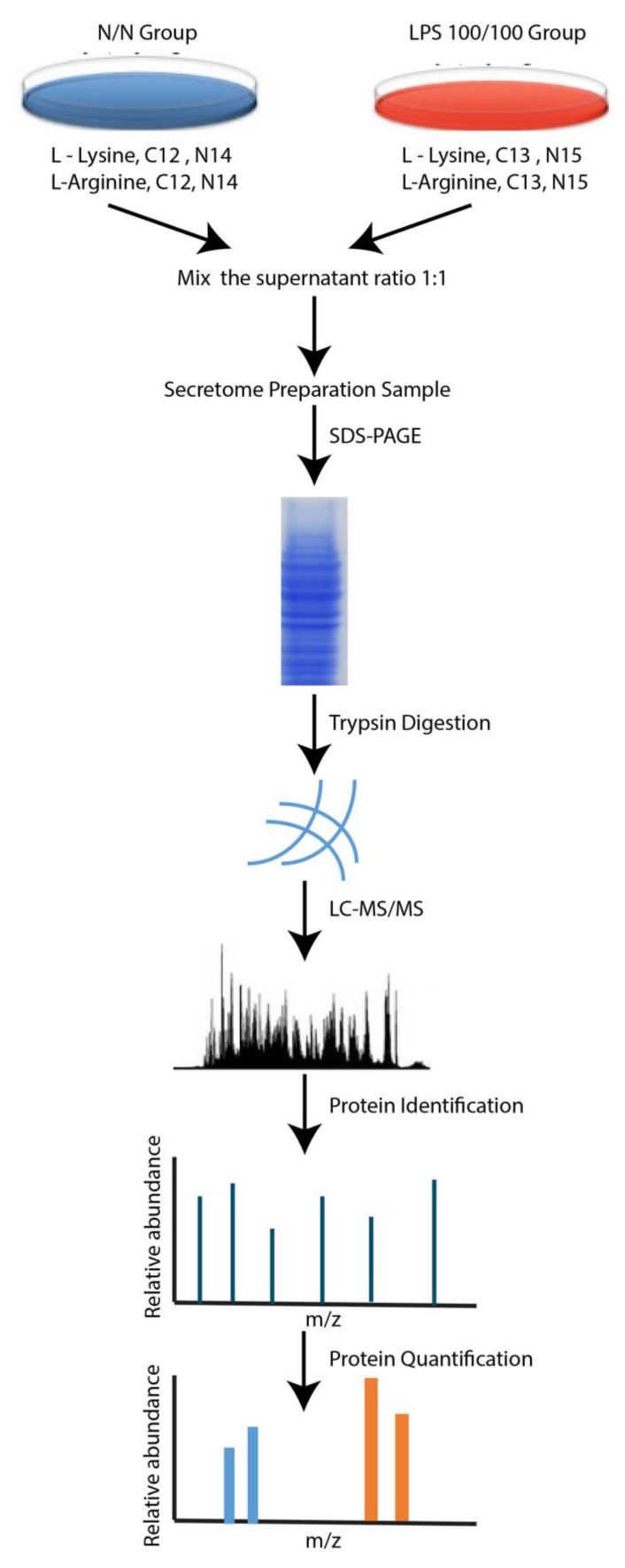
Diagram of the secretome analysis procedure.

A mouse monocyte-macrophage (RAW264.7) cell line was cultured in complete Dulbecco’s modified Eagle’s medium (cDMEM) (Thermo Fisher Scientific, Waltham, MA, USA). Stable isotope labeling with amino acids in cell culture (SILAC) was performed using heavy isotope-labeled lysine and arginine (Cambridge Isotopes Laboratories, Inc., Tewksbury, MA, USA). LPS tolerance (LPS/LPS) was induced with LPS at 100 ng/well with macrophages at 1 × 10^5^ cells/well for 24 h. Then the cells were washed and re-stimulated with the same dose of LPS. Cells in this group were grown in cDMEM labeled with Arg^+10^ and Lys^+8^. In parallel, the cells from the control group were grown in cDMEM with Arg^0^ and Lys^0^ and washed with cDMEM instead of LPS (N/N). The media was collected at 24 h post-second dose of LPS (or second cDMEM), centrifuged for debris separation, dried in a vacuum centrifuge (SpeedVac, Thermo Fisher Scientific) and prepared for the secretome analysis essentially as described [22]. Briefly, the dried secreted proteins were resuspended in 2× SDS-PAGE loading buffer, separated using a 10% Bis-Tris NuPAGE gel with 3-(N-morpholino)propanesulfonic acid (MOPS) buffer and run at 200 V for 40 min. Then the gel was fixed, washed, stained with PageBlue protein staining solution (Thermo Fisher Scientific) and destained with ddH_2_O overnight at 4 °C. After that the lanes were cut from the gel using razor blades before the in-gel tryptic digestion according to the published method [23]. Subsequently, the peptides were analyzed on a Q Exactive HF mass spectrometer (MS) and MS files were processed with Proteome Discoverer^TM^ software version 2.1 (Thermo Fisher Scientific) and searched with the SEQUEST-HT search engine against a mouse UniProt FASTA database The following parameters were set for the search: (1) digestion enzyme: trypsin; (2) maximum allowance for missed cleavages: 2; (3) maximum of modifications: 4; (4) fixed modifications: carbamidomethylation of cysteine (57.02146 Da); (5) variable modifications: oxidation of methionine (15.99491 Da) and light (Arg, Lys) and heavy (Arg +10.00827, Lys +8.01420) isotope labeling. The control (N/N) channels were used as denominators to generate abundance ratios of LPS tolerance (LPS/LPS)/control (N/N). Significantly differentially regulated proteins were determined by Mann–Whitney U test with a *p* value of 0.05 considered significant. Bioinformatics: Signaling pathway analysis for the proteins identified was performed with the tools available at the Database for Annotation, Visualization and Integrated Discovery (DAVID, v6.8, http://david.abcc.ncifcrf.gov/) and Kyoto Encyclopedia of Genes and Genomes (KEGG) (http://www.genome.jp/kegg/pathway.html) [24]. The STRING online software (version 10.5) was used to search for protein interactions between the identified proteins and a required confidence (combined score) of >0.9 was used as the cut-off criterion [25]. The short diagram of the procedures for secretome analysis was demonstrated in Figure 1.

### 2.3. Western Blot Analysis and NFκB Detection in RAW264.7 Cells

Western blot analysis on RAW264.7 cells was performed according to the previous publication [14] with rabbit polyclonal antibody against 24p3R (Abcam, Cambridge, MA, USA) and antibody against glyceraldehyde 3-phosphate dehydrogenase (GAPDH) (Abcam). To evaluate the effect of LCN2 on NFκB signaling [26], RAW264.7 cell (1 × 10^5^ cells/well) with protocol of a single LPS stimulation (N/LPS), LPS tolerance induction (LPS/LPS) and control (N/N) were stimulated with 140 µM/well of rLcn-2 and incubated at 37 °C in 5% CO_2_ for 24 h prior to collection. Then 400 µL/well of TRIzol (Invitrogen, Carlsbad, CA, USA) was used to extract cellular RNA and performed quantitative polymerase chain reaction (qPCR) with the nucleotide sequences primer of mouse *NFκB* RelA; forward 5′-CTTCCTCAGCCATGG TACCTCT-3′, reverse 3′-CAAGTCTTCATCAGCATCAAACTG-5′ and normalized to the expression of β-actin with the 2−ΔΔCT method [27].

### 2.4. Cell Viability and Metabolic Activity of RAW264.7 Cells

The cell viability of RAW264.7 cells after stimulation was determined by nuclear staining with Hoechst 33342 dye (Thermo Fisher Scientific) at 10 µg/mL (in phosphate buffer solution: PBS) with 15 min incubation at 37 °C in the dark before washing with PBS and photographed by an IX81 inverted microscope (Olympus, Tokyo, Japan). The viable cells (blue colored dots) were counted against area determination using ImageJ (NIH, Bethesda, MD, USA). In addition, the metabolic activity of cells after stimulation was analyzed by the capability of reducing the tetrazolium dye 3-(4,5-dimethylthiazol-2-yl)-2,5-diphenyltetrazolium bromide (MTT assay). The cells were incubated with 0.5 mg/mL of MTT solution (Thermo Fisher Scientific) for 2 h at 37 °C in the dark. Following removal of the MTT from the wells, the MTT was diluted with DMSO then wrapped to avoid light during shaking for 5 min. Finally, the reduction of the MTT was measured using a Varioskan Flash microplate reader (Thermo Fisher Scientific) at absorbance 570 nm.

### 2.5. Supernatant Cytokines, Lcn-2 and Extracellular Flux Analysis

Supernatants from the RAW264.7 cells after a single LPS stimulation (N/LPS), LPS tolerance induction (LPS/LPS), and control cells (N/N) were collected at the specific time-points for cytokines and Lcn-2 measurements (ELISA, Quantikine R&D systems). In addition, the energy metabolism profiles with the estimation of glycolysis and mitochondrial oxidative phosphorylation with extracellular acidification rate (ECAR) and oxygen consumption rate (OCR) were performed, respectively, by Seahorse XF Analyzers (Agilent, Santa Clara, CA, USA) as previously described [28]. RAW264.7 cells in different experimental groups were dispersed into a monolayer for the measurement.

### 2.6. Lipocalin-2 Induction, Recombinant Lcn-2 Stimulation and Anti-Lcn-2 Blocking Agent in RAW264.7 Cells

RAW264.7 cells (1 × 10^5^ cells/well) were treated with cDMEM for 24 h then washed and incubated with mouse recombinant cytokines (TNF-α, IL-6 and IL-10) (R&D systems) at dose 50 ng/well or LPS (N/LPS) at 100 ng/well or starved by incubation with Earle’s balanced salt solution (Gibco, Grand Island, NY, USA), to compare with LPS tolerance (LPS/LPS), and incubated at 37 °C in 5% CO_2_ before supernatant collection for Lcn-2 analysis. In addition, Lcn-2 stimulation by recombinant Lcn-2 (rLcn-2) at 140 µM/well was compared to Lcn-2 blocking by anti-Lcn-2 antibody (ThermoFisher Scientific) at 50 µg/well [29] was incubated with LPS (in N/LPS) or the second dose of LPS (in LPS/LPS) or the second cDMEM (in control N/N) before determination of supernatant cytokine levels by ELISA (Quantikine).

Moreover, the evaluation of macrophage polarization in RAW264.7 cell (1 × 10^5^ cells/well) was performed. Briefly, the cells were incubated with LPS (10 ng/well; M1 polarization stimulator), IL-4 (20 ng/well; M2 polarization inducer) or rLcn-2 (140 µM/well) or control cDMEM at 37 °C in 5% CO_2_ for 24 h before supernatant removal and addition of 400 µL/well of TRIzol (Invitrogen) to extract cellular RNA. Then quantitative polymerase chain reaction (qPCR) was performed for the characteristics of M1 (*iNOS*, *TNF-a* and *IL-1β*) and M2 (*Arginase-1*, *IL-10* and *TGF-β*). The nucleotide sequences were *iNOS*, forward, 5′-CCCTTCCGAAGTTTCTGGCAGCAGC-3′, reverse: 5′-GGCTGTCAGAGCCTCGTGGCTTTG-3′; *Arginase-1*, forward, 5′-CAGAAGAATG GAAGAGTCAG-3′, reverse: 5′-CAGATATGCA GGGA GTCACC-3′; *TNF-a*, forward, 5′-CCTCACACTCAGATCATCTTCTC-3′, reverse: 5′-AGATCCATGCCG TTGGCCAG-3′; *IL-1β,* forward, 5′-GAAATGCCACCTTTTGACAGTG-3′, reverse: 5′-TGGATGCTCTCATCAGGACAG-3′; *IL-10*, forward, 5′-GCTCTTACTGACTGGCATGAG-3′, reverse: 5′-CGCAGCTCTAGGAGCATGTG-3′; *TGF-β*, forward, 5′-CAGAGCTGCGCTTGCAGAG-3′, reverse: 5′-GTCAGCAGCCGGTTACCAAG-3′. The expression of each gene was normalized to the expression of β-actin using the 2−ΔΔCT method [30,31,32,33].

### 2.7. Bone Marrow Derived Macrophages, Granulocyte-Monocyte Colony Stimulating Factor (GM-CSF) and Recombinant Lcn-2

Bone marrow (BM) derived macrophages from WT and FcGRIIb-/- mice were cultured following a protocol described elsewhere [11,14] and then stimulated to induce LPS tolerance (LPS/LPS) or single LPS dose (N/LPS) for supernatant collection. To examine the alteration of Lcn-2 after attenuation of LPS tolerance, granulocyte-monocyte colony stimulating factor (GM-CSF) (R&D systems) at 70 ng/well [34] or rLcn-2 at 140 µM /well was incubated together with the second dose of LPS before supernatant collection.

### 2.8. Statistical Analysis

All statistical analyses were performed with GraphPad Prism 5.0 (GraphPad Software, Inc., San Diego, CA, USA). The differences between groups were examined by Student’s *t*-test or one-way analysis of variance (ANOVA) followed by Bonferroni analysis for the comparison of two or more experimental groups. The in vitro experiments were based on the independent triplicate experiments and were represented by mean ± standard error (SE) where *p* value <0.05 was considered statistically significant.

## 3. Results

### 3.1. LPS Tolerance Enhanced Lcn-2 in Macrophages, Despite the Energy Depletion

**Figure 2:**
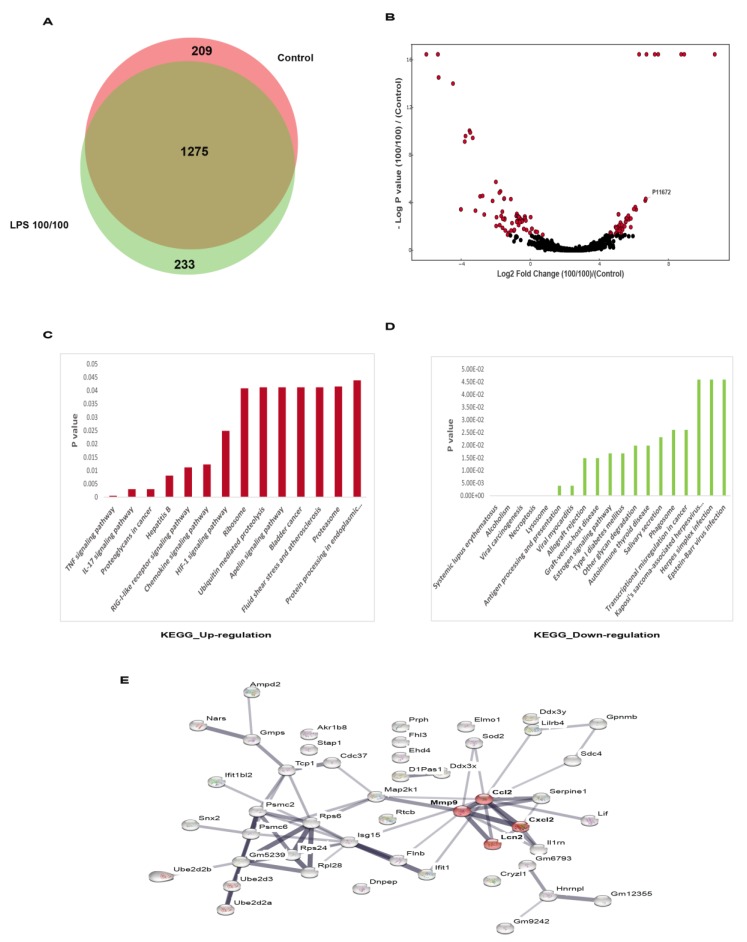
The volcano plot analysis of proteomic from supernatant of RAW264.7 cells after sequential lipopolysaccharide (LPS) stimulation (LPS tolerance; LPS/LPS) compared with control (N/N) (see method) demonstrated down- and up-regulated proteins (**A**) and the Venn diagram indicated the different number of proteins from LPS tolerance (LPS/LPS) group versus the control (**B**). Additionally, pathway analysis clusters (DAVID) of up- and down-regulated proteins (**C**,**D**) and the enriched pathway of the proteins in LPS/LPS compared with the control (**E**) were demonstrated.

Because (i) the insufficient cytokine production during LPS tolerance deteriorates organismal control [11] and (ii) the secretome changes during LPS tolerance could be significant, secretome analysis in RAW264.7 cells with LPS tolerance or control was performed using mass spectrometry. Among the total number of 1707 identified proteins, 209 and 233 proteins unique to the control or LPS-tolerant RAW264.7 cells were identified, as shown in the Venn diagram (Figure 2A). The volcano plot was constructed to graphically display the quantitative analysis of the proteins with higher abundance (right side of the graph) and lower abundance (left side of the graph) in the LPS tolerant cells compared to control (*p* < 0.05 by the student *t*-test) (Figure 2B).

In addition, the functional analysis of the up- and down-regulated proteins was performed using the KEGG database (Figure 2C,D). While the downregulation of several cytokines has been frequently mentioned in LPS tolerant macrophages, the up-regulated proteins have been relatively less studied. The protein-protein interaction analysis of the up-regulated proteins with the STRING online software demonstrated the interactions between Lcn-2, Matrix metallopeptidase 9 (MMP-9), chemokine (C-C motif) ligand 2 (CCL-2) and chemokine (C-X-C motif) ligand 2 (CXCL-2) (Figure 2D). Among these proteins, the functions of chemokines (CCL-2, CXCL-2) and collagenase protein (MMP-9) have been already mentioned in this context [35], but the impact of Lcn-2 on LPS tolerance was not known.

**Figure 3:**
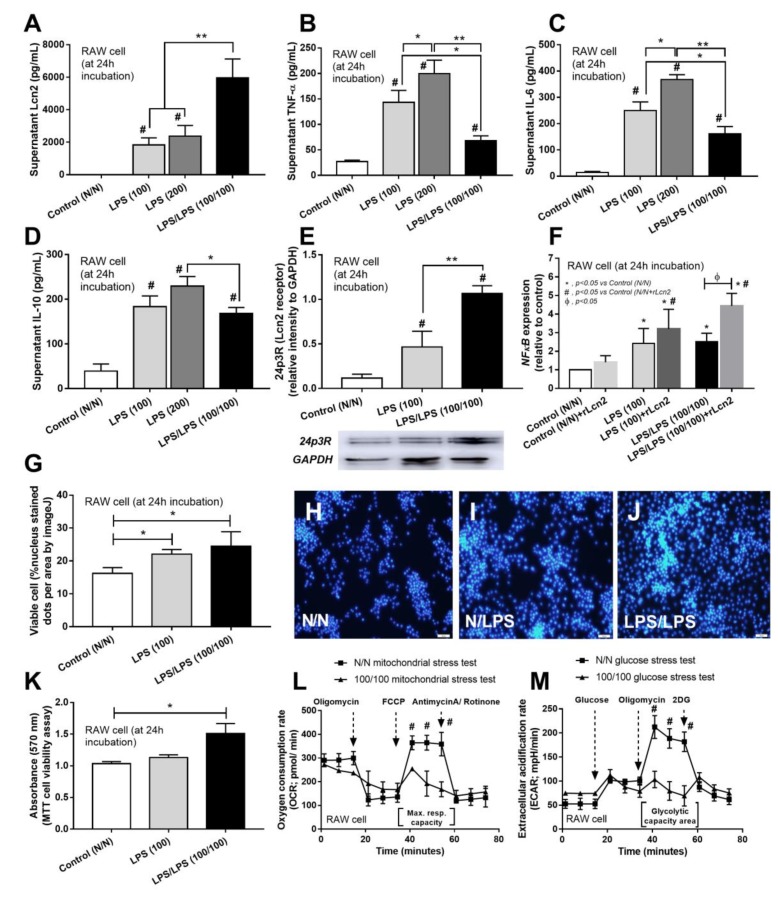
The characteristic of RAW264.7 cell after LPS stimulation once (two doses; 100 and 200 ng/well) or twice, LPS/LPS (100/100), versus control (N/N) (see method) as determined by supernatant cytokines (**A**–**C**) and Lipocalin-2 (Lcn-2; **D**) were demonstrated. Additionally, Lcn-2 receptor (24p3R) after cell stimulations by Western blot analysis (**E**) and NFκB, a possible downstream signaling of Lcn-2, in cells with or without recombinant Lcn-2 (rLcn-2) activation by relative mRNA expression (**F**) were indicated. Moreover, cell viability as determined by nuclear staining as calculated by ImageJ (**G**), representative nuclear stain figures (**H**–**J**) and metabolic activity (MTT assay, see method) (**K**) (see method) were determined. Further, extracellular flux analysis of RAW264.7 cell with LPS/LPS versus control as demonstrated by oxygen consumption rate of mitochondria stress test (**L**) and extra-cellular acidification rate of glucose stress test (**M**) were demonstrated. (Independent triplicate experiments were performed for all experiments; *, *p* < 0.05; **, *p* < 0.01; #, *p* < 0.01 vs. N/N); Max. Resp. Capacity, maximum respiratory capacity area.

ELISA analysis of supernatants of LPS-tolerant cells confirmed decreased cytokine secretion (shown for TNF-α, IL-6 and IL-10) but increased Lcn-2 in comparison with the single LPS-stimulated (LPS) or control (N/N) RAW264.7 cells (Figure 3A–D). In addition, increased accumulation of receptor of Lcn-2 (24p3 receptor) was demonstrated by Western blot analysis following LPS stimulation, both with single and sequential doses, but it was more prominent following the sequential LPS tolerance protocol (Figure 3E). Because NFκB is a transcriptional factor for several cytokines production and is one of the down-regulation signals of LPS stimulation [36], rLcn-2 stimulation was used to test if NFκB is also downstream of rLcn-2. Indeed, NFκB increased similarly between either single or double LPS stimulation protocols but the addition of rLcn-2 enhanced NFκB expression more predominantly with LPS tolerance (Figure 3F).

A possible cause for the changing accumulations of specific cytokines could be changes in cell viability or metabolic activity. To account for this, the number of viable cells after LPS stimulation was tested. As such, enhanced cell viability and metabolic activity, were determined by nucleus staining and MTT assay, respectively, in macrophages with both LPS stimulation protocols, (Figure 3G–K). These results confirmed that the decreased production of cytokines by LPS tolerant cells was not due to decreased cell viability or overall metabolic activity. Additionally, while overall cellular metabolic activity, as measured by the MTT assay, increased in the LPS tolerant cells versus the control cells, Seahorse analysis showed that LPS tolerance depleted the cellular energy observed both in mitochondria and glucose stress tests (Figure 3L,M). Interestingly, despite the depletion of cellular energy, increased Lcn-2 production occurred suggesting an important role of Lcn-2 in LPS tolerance.

**Figure 4:**
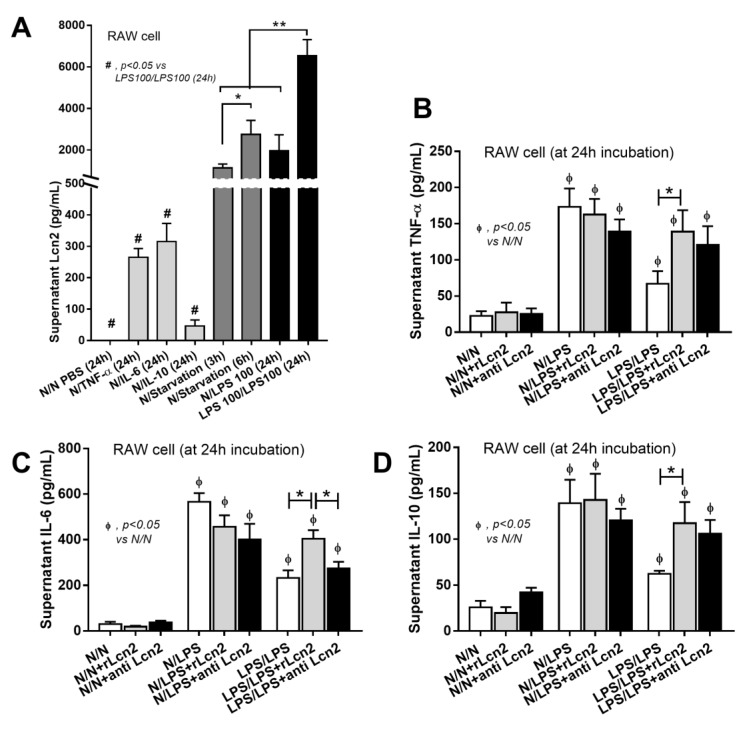
The induction of Lipocalin-2 (Lcn-2) (**A**) in supernatant of RAW264.7 cell with several activators including cytokines (TNF-α, IL-6 and IL-10) (**B**–**D**) for 24 h, starvation (for 3 h and 6 h), LPS (for 24 h) and LPS tolerance (LPS/LPS at 24 h post-second dose of LPS) (see method) (**A**) were demonstrated. Additionally, the impact of recombinant Lcn-2 (rLcn-2) or LCN-2 blocking antibody (anti-Lcn-2) in RAW264.7 cell after LPS stimulation once (N/LPS) or twice, LPS/LPS versus control (N/N) as determined by supernatant cytokines were demonstrated. (Independent triplicate experiments were performed for all experiments; *, *p* < 0.05; **, *p* < 0.01; #, *p* < 0.001).

**Figure 5:**
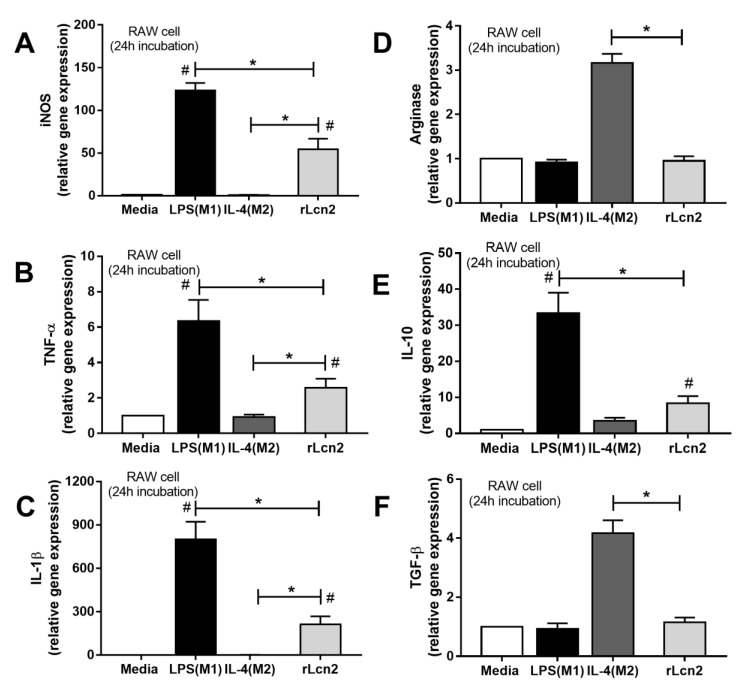
The evaluation of macrophage polarization after incubation with LPS (M1 induction), IL-4 (M2 induction), recombinant Lcn-2 (rLcn-2) and media control (Media) in RAW264.7 cell as determined by relative mRNA expression in markers of M1 (*iNOS*, *TNF-α* and *IL-1β*) (**A**–**C**) and M2 polarization (*Arginase-1*, *IL-10* and *TGF-β*) (**D**–**F**) was demonstrated. (Independent triplicate experiments were performed for all experiments; *, *p* < 0.05).

In addition, LPS tolerance was the most potent Lcn-2 trigger of all the stimulants used (cytokines, starvation and single LPS incubation) (Figure 4A) and treatment with recombinant Lcn-2 (rLcn-2) enhanced cytokine production only in sequential LPS stimulations (Figure 4B–D). Further, blocking Lcn-2 with anti-Lcn-2 reduced IL-6 production in LPS tolerant cells but not cells following single LPS stimulation (Figure 4B–D) suggesting the importance of Lcn-2 in LPS tolerance. Also, rLcn-2 drove macrophage toward inflammatory M1 polarization (increased *iNOS*, *TNF-α* and *IL-1β*) more strongly than to anti-inflammatory M2 polarization (increased *IL-10* but not *Arginase 1* nor *TGF-β*) (Figure 5). The totality of these data suggests that Lcn-2 might be secreted to counteract the cellular inertia in the LPS tolerance.

### 3.2. Prominent LPS Tolerance, but No Difference in Lcn-2 Production, in FcGRIIb-/- Macrophages in Comparison with Wild-Type Cells

**Figure 6:**
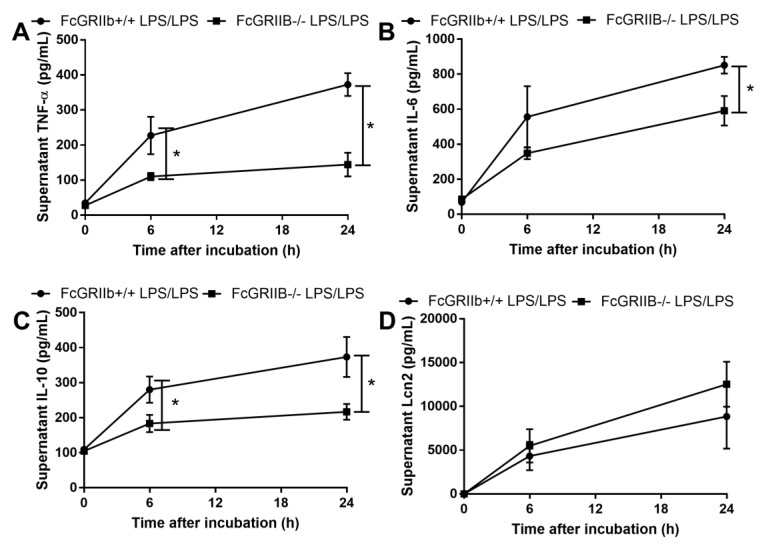
The characteristics of bone marrow derived macrophage from wild-type (FcGRIIb+/+) and FcGRIIb-/- mice with double LPS stimulation (LPS/LPS; LPS tolerance) as determined by supernatant cytokines (**A**–**C**) and Lipocalin-2 (Lcn-2) (**D**) were demonstrated. (Independent triplicate experiments were performed for all experiments; *, *p* < 0.05).

**Figure 7:**
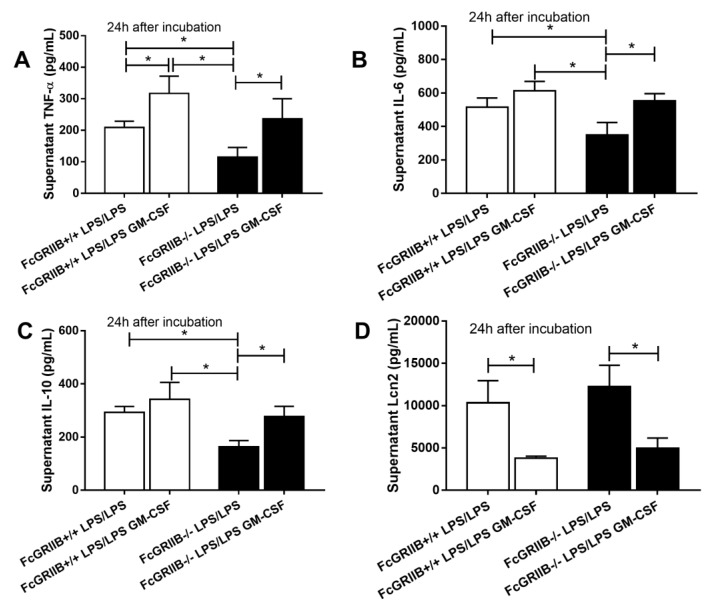
The impact of granulocyte-monocyte colony stimulating factor (GM-CSF) against LPS tolerance (double LPS stimulation; LPS/LPS) in wild-type (WT) and FcGRIIb-/- macrophage as determined by supernatant cytokines (**A**–**C**) and Lipocalin-2 (Lcn-2) (**D**) were demonstrated. (Independent triplicate experiments were performed for all experiments; *, *p* < 0.05).

**Figure 8:**
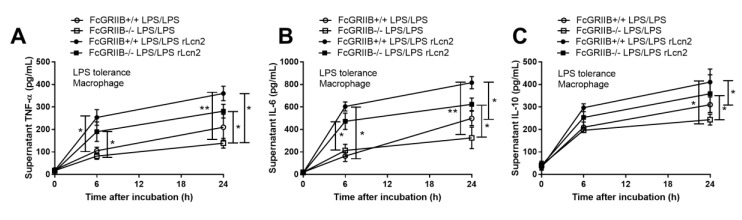
The impact of recombinant Lcn-2 (rLcn-2) against LPS tolerance (double LPS stimulation; LPS/LPS) in wild-type (WT) and FcGRIIb-/- macrophage as determined by supernatant cytokines (**A**–**C**) were demonstrated. (Independent triplicate experiments were performed for all experiments; *, *p* < 0.05; **, *p* < 0.01).

Due to the loss of inhibitory signaling of FcGRIIb-/- macrophages, higher supernatant cytokine levels after single LPS stimulation (LPS hyper-responsiveness) and lower cytokine production after the induction of LPS tolerance (vigorous LPS exhaustion) in FcGRIIb-/- compared with wild-type (WT) cell was observed (Supplemental Figure S1A–C) as previously mentioned [11,14]. Interestingly, the supernatant Lcn-2 level in LPS-tolerant FcGRIIb-/- and WT macrophages was not different (Figure 6D). However, single LPS stimulation increased Lcn-2 secretion more profoundly in FcGRIIb-/- than in WT cells (Supplemental Figure S1D). Because the role of secreted Lcn-2 by macrophage after LPS tolerance is still unclear, whether to further induce the more severe LPS tolerance or as a counter effect against the tolerance, the direction of Lcn-2 level after the attenuation LPS tolerance might be informative. GM-CSF was used to attenuate LPS tolerance [34,37], producing a more prominent effect in FcGRIIb-/- macrophages with an increase in all supernatant cytokines in FcGRIIb-/- versus increase only TNF-α in WT cells (Figure 7A–C). GM-CSF also reduced supernatant Lcn-2 in both cell-types (Figure 7D), suggesting increased Lcn-2 production was in response to LPS tolerance. As such, rLcn-2 attenuated LPS tolerance in both cell types as shown for all the selected cytokines (Figure 8A–C). These results suggest inadequate Lcn-2 production in FcGRIIb-/- macrophages affect LPS tolerance.

**Figure 9:**
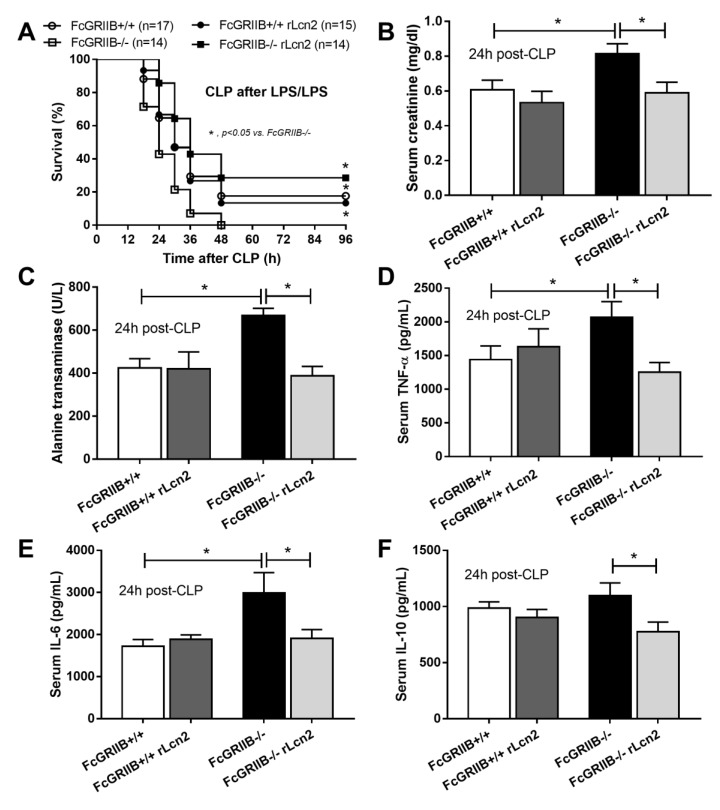
The characteristics of cecal ligation and puncture sepsis surgery after LPS tolerance induction by sequential LPS stimulation (CLP after LPS/LPS) (see method) in wild-type (WT) and FcGRIIb-/- mice with and without recombinant Lcn-2 (rLcn-2) administration as evaluated by survival analysis (**A**) and serum markers of injury including serum creatinine (**B**), alanine transaminase (**C**) and cytokines (**D**–**F**) (*n* = 7–10/group for B–F) were demonstrated. *, *p* < 0.05.

Because rLcn-2 attenuated LPS tolerance (Figure 8) and LPS tolerance worsens the severity of bacterial sepsis through the defect on organismal control [11], the importance of LPS tolerance in the sepsis mouse model by pre-conditioning with LPS tolerance before cecal ligation and puncture surgery (LPS-CLP) was tested. The mortality rate in LPS-CLP of FcGRIIb-/- mice was more severe than WT (Figure 9A). Interestingly, rLcn-2 administration reduced sepsis mortality rate only in FcGRIIb-/- mice with the LPS-CLP procedures, but not in WT (Figure 9A), despite the attenuation of LPS tolerance on macrophages from both strains (Figure 8). In addition, rLcn-2 improved renal function, reduced liver injury and attenuated serum cytokines only in FcGRIIb-/- mice (Figure 9B–F) suggesting the different in Lcn-2 requirement between WT and FcGRIIb-/- lupus mice. On the other hand, rLcn-2 could not attenuate CLP without LPS preconditioning in the WT and FcGRIIb-/- mice (data not shown).

## 4. Discussion

Proteomic analysis of the supernatants from LPS-tolerant RAW264.7 cells identified Lcn-2 among the upregulated proteins. The administration of recombinant Lcn-2 (rLcn-2) attenuated LPS tolerance, causing the activation toward inflammatory responses. In contrast to other cytokines, the level of supernatant Lcn-2 in LPS-tolerant FcGRIIb-/- macrophages was similar to LPS-tolerant WT cells, despite the more severe LPS exhaustion, as seen by the decreased levels of many cytokines, typical of FcGRIIb-/- macrophages. These results indicate a defect in Lcn-2 production by FcGRIIb-/- macrophages. Indeed, the introduction of recombinant Lcn-2 attenuated LPS exhaustion and improved sepsis response of the FcGRIIb-/- lupus mice in the LPS pre-conditioning CLP model.

### 4.1. LPS Tolerance Induced Energy Depletion and Lcn-2 Secretion as an Autocrine Signal

Although LPS tolerance induced the proliferation of RAW264.7 cells in comparison with non-LPS stimulated controls, supernatant cytokines were lower than a single LPS stimulation. Decreased production of supernatant cytokines in LPS-tolerant RAW264.7 cells was, at least in part, responsible for the defect in energy production both in mitochondrial and glycolytic pathways, as determined by extracellular flux analysis. Interestingly, a high amount of Lcn-2 and Lcn-2 receptor were produced in LPS tolerant cells despite the severe energy-depletion in macrophages implying an important role of Lcn-2 in LPS tolerance. Although Lcn-2 is primarily responsible for the cellular iron homeostasis and trafficking [38], other roles of Lcn-2 [39] including inflammatory responses and hepatocyte lipid metabolism through several downstream signaling, including NFκB [26], are mentioned. Here, the addition of rLcn-2 in single or double LPS stimulation conditions enhanced NFκB expression versus either condition in the absence of rLcn-2, supporting the role of Lcn-2 in the downstream regulation of NFκB signaling [26].

Because LPS tolerance is an alternative state of M2 polarization [40], the induction toward M1 polarization (a pro-inflammatory stage) by Lcn-2, as previously mentioned [41], might be beneficial. Indeed, rLcn-2 attenuated LPS tolerance (increased levels of the supernatant cytokines), while the inhibition of Lcn-2 worsened LPS tolerance (reduced levels of the supernatant IL-6). The enhanced inflammatory responses caused by Lcn-2, also referred to as Neutrophil Gelatinase-Associated Lipocalin (NGAL), have been demonstrated in several conditions [26,42,43]. However, rLcn-2 did not enhance cytokine levels in RAW264.7 cells with a single LPS stimulation, possibly due to the strong pro-inflammatory potency of LPS in macrophage activation. Hence, the pro-inflammatory action of Lcn-2 could not prevail over the effect of LPS.

In contrast, Lcn-2 should be beneficial in late-immune exhaustion phase of sepsis [44]. Because secondary infection during the immune exhaustion in sepsis is caused, in part, by LPS tolerance [45] and the resulting inadequate inflammatory cytokine release dampening organismal control [11], the attenuation of sepsis immune exhaustion might be beneficial. It is interesting to note that a single LPS stimulation also activates Lcn-2 production from macrophages but at a lower level than the stimulation inducing LPS tolerance, suggesting the weaker role of Lcn-2 in the acute inflammatory state. Hence, our results from RAW264.7 cells suggest that macrophages secrete Lcn-2 to counteract LPS tolerance.

### 4.2. Prominent LPS Tolerance but Inadequate Lcn-2 Production in FcGRIIb-/- Macrophages

Despite the more severe LPS exhaustion in FcGRIIb-/- macrophages, Lcn-2 production was not different between FcGRIIb-/- and WT cells. Additionally, GM-CSF attenuated LPS tolerance in FcGRIIb-/- cells (increased all three measured cytokine levels) but reduced Lcn-2 secretion suggesting that Lcn-2 was not necessary for the GM-CSF treated cells. Moreover, the role of Lcn-2 as a countersignal against LPS tolerance was supported by the attenuation of LPS tolerance in FcGRIIb-/- (and WT) cells incubated with rLcn-2. Although the dampened cytokine response in LPS tolerance is preventing the host from the lethal response, the limited cytokine response worsens the secondary bacterial infection as cytokines are necessary for the organismal control [11]. Indeed, rLcn-2 attenuated sepsis in the CLP with LPS tolerance in the FcGRIIb-/- mice but not in the WT mice. This possibly correlated with the inadequacy of Lcn-2 to counter severe LPS exhaustion in the FcGRIIb-/- mice. In parallel, Lcn-2 in the WT mice might be already adequate to curb the less severe LPS exhaustion so the administration of rLcn-2 was not effective in sepsis attenuation in WT mice. However, the effectiveness of rLcn-2 in the treatment of LPS tolerance in lupus mice was interesting because an active lupus condition could induce intestinal barrier impairment, spontaneous endotoxemia, LPS tolerance and enhanced bacterial susceptibility [5,6]. Hence, we propose the use of Lcn-2 to remedy LPS tolerance especially in lupus with bacterial infection. Further studies on the identification of biomarkers for LPS tolerance and Lcn-2 administration in lupus are needed.

In conclusion, Lcn-2 was produced by LPS-tolerant macrophages, despite the severe energy depletion, possibly as an autocrine signal against LPS tolerance. Higher Lcn-2 levels might be necessary for FcGRIIb-/- macrophages (and mice) to counteract the more severe LPS tolerance compared with WT. The administration of rLcn-2 neutralized the LPS exhaustion in lupus mice, but not in WT mice, and attenuated the secondary bacterial infection after LPS tolerance. Hence, rLcn-2 can be considered as a candidate drug for LPS tolerance in patients with lupus, especially with recurrent infections.

## Supplementary Materials

The following are available online at https://www.mdpi.com/2073-4409/8/9/1064/s1, Figure S1: The characteristics of bone marrow derived macrophage from wild-type (FcGRIIb+/+) and FcGRIIb-/- mice with once LPS stimulation (N/LPS) as determined by supernatant cytokines (A-C) and Lipocalin-2 (Lcn-2).
