# Supplementary material for: Lipocalin-2 (Lcn-2) Attenuates Polymicrobial Sepsis with LPS Preconditioning (LPS Tolerance) in FcGRIIb Deficient Lupus Mice
Source: Cells. 2019 Sep 11;8(9):1064. doi: 10.3390/cells8091064 (PMC6769833; doi:10.3390/cells8091064)
Supplement: Supplementary file 1 [file cells-08-01064-s001.pdf]

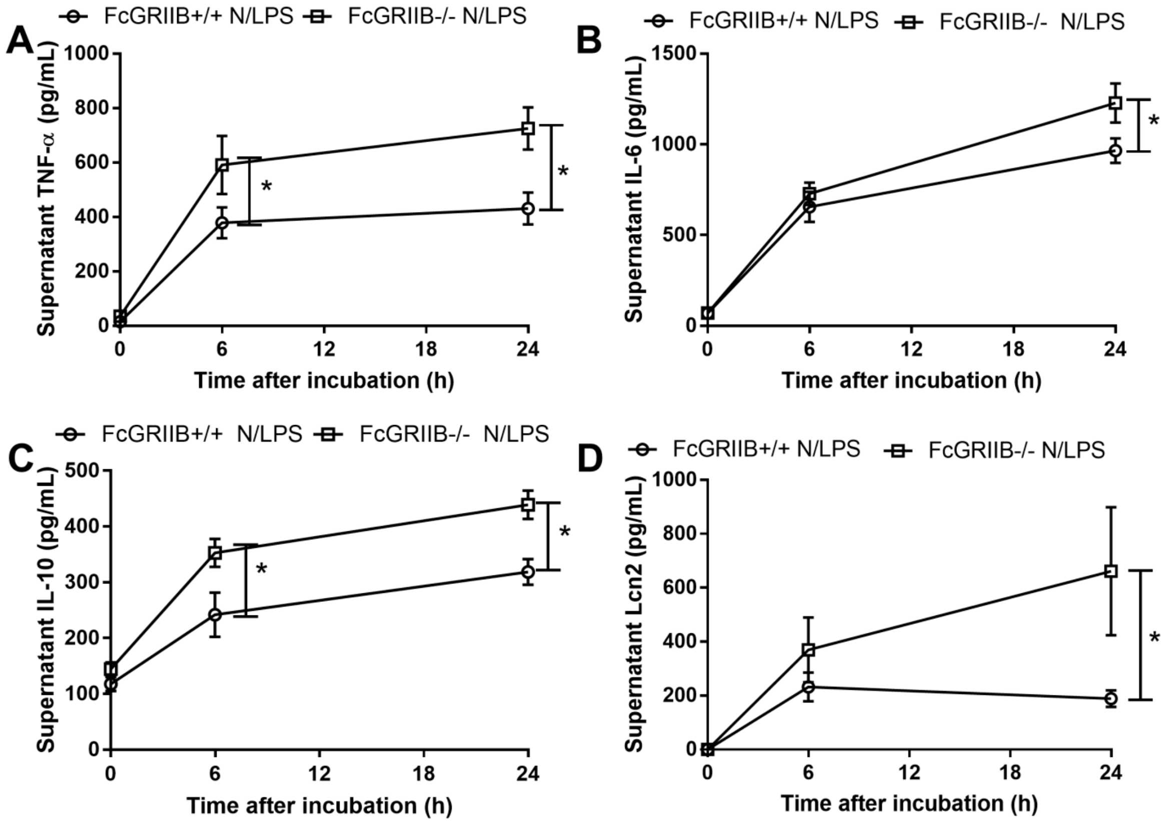

Figure S1. The characteristics of bone marrow derived macrophage from wild-type (FcGRIIb+/+) and FcGRIIb-/- mice with once LPS stimulation (N/LPS) as determined by supernatant cytokines (A-C) and Lipocalin-2 (Lcn-2) were demonstrated. (Independent triplicate experiments were performed for all experiments; *, $p<0.05$).
